# Cortical alterations in patients with mild cognitive impairment who converted to Alzheimer disease compared to non-converters and correlations of these alterations with mild behavioral impairment

**DOI:** 10.1192/j.eurpsy.2025.1668

**Published:** 2025-08-26

**Authors:** A. S. Tomyshev, N. S. Cherkasov, A. Y. Komarova, E. G. Abdullina, I. V. Kolykhalov, I. S. Lebedeva

**Affiliations:** 1 Laboratory of neuroimaging and multimodal analysis; 2 Department of gerontological psychiatry, Mental Health Research Center, Moscow, Russian Federation

## Abstract

**Introduction:**

There is a growing evidence that a presence of mild behavioral impairment (MBI) in geriatric patients with mild cognitive impairment (MCI) increases the risk of Alzheimer disease. However, neurobiological patterns underling such additional risk remains unclear.

**Objectives:**

We aimed to investigate structural cortical patterns in MCI patients that differentiate converters from non-converters to Alzheimer disease (AD) and to explore correlations of such patterns with mild behavioral impairment.

**Methods:**

Thirty five right-handed geriatric patients with amnestic type of MCI (aMCI) were followed up during the period of 11.3±6.9 months and divided into converters to AD (n=11, mean age 74.6±7.5, 10 females) and non-converters (n=24, mean age 72.6±8.1, 18 females). Patients and matched healthy controls (n=17, mean age 72.3±7.2 years, 14 females) underwent structural 3T MRI at baseline. MRI images were processed via FreeSurfer 6.0 to quantify gray matter thickness for 68 cortical areas according to Desikan atlas. Cognitive status was assessed using the Montreal Cognitive Assessment (MoCA) scale, and the severity of mild behavioral impairment was assessed using the MBI-C (Mild Behavioral Impairment Checklist) at baseline.

**Results:**

Cortical thickness in the left inferior parietal lobe (IPL) and left middle temporal gyrus (MTG) were decreased in converters compared to both non-converters (IPL: F(1,30)=12.8, p=0.0012, Cohen’s *d*=−1.30; MTG: F(1,30)=12.8, p=0.0012; Cohen’s *d*=−1.30) and healthy controls (IPL: F(1,24)=11.4, p=0.0025, Cohen’s *d*=−1.33; MTG: F(1,24)=8.3, p=0.008; Cohen’s *d*=−1.15) (Image 1A,B).

Converters also showed larger baseline MBI-C scores compared to non-converters (13.8±12.0 vs 7.8±6.6; GML t=2.1, p=0.045) and no difference in baseline MoCA (21.8±4.0 vs 23.2±2.8; GML t=−1.4, p=0.19).

Baseline MBI-C scores in the whole aMCI group correlated negatively with cortical thickness both in the left IPL (R=−0.53, p=0.0011) and left MTG (R=−0.48, p=0.004) (Image 1C). No correlations between MoCA scores and cortical thickness were observed.

Image 1. A: Clusters of decreased cortical thickness according to atlas of Desikan et al. (2006) in converters compared to non-converters and healthy controls. B: Box-plots of cortical thickness in the left inferior parietal lobe and left middle temporal gyrus. C: Scatter plots of MBI-C scores and gray matter thickness in the left inferior parietal lobe and left middle temporal gyrus in the whole aMCI group (n = 35).

**Image 1:**

**Figure:**
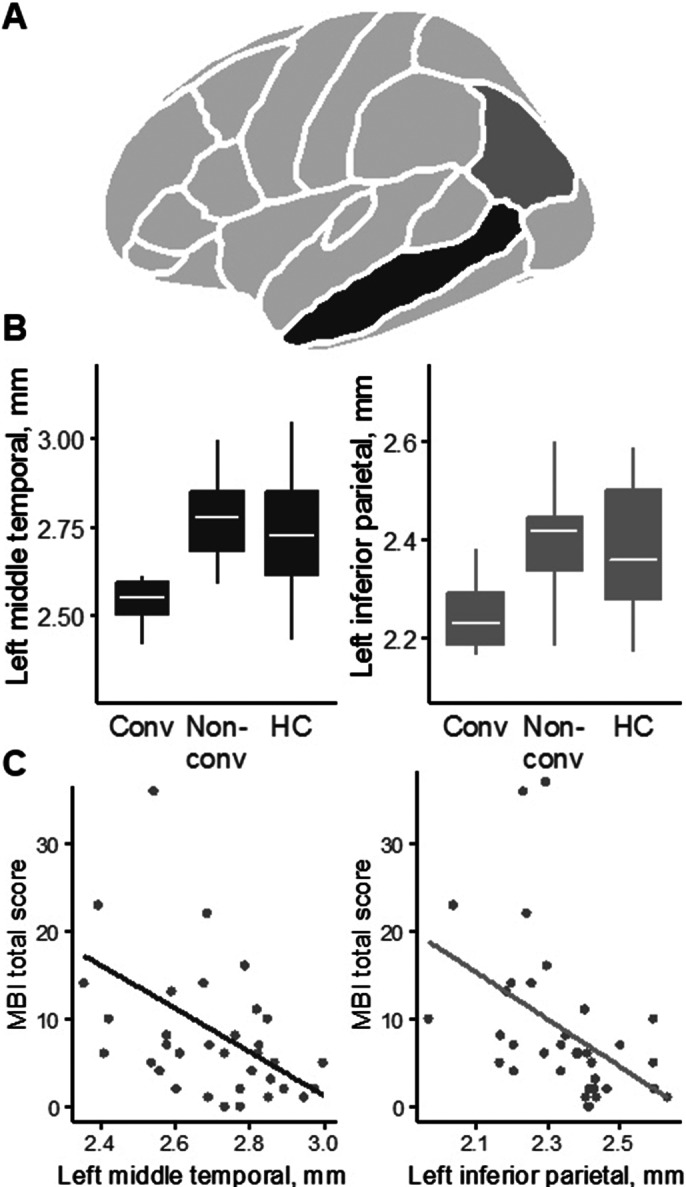

**Conclusions:**

The findings suggest that the decreased baseline cortical thickness in the left inferior parietal lobe and middle temporal gyrus with associated greater severity of mild behavioral impairment could be potential marker of increased risk of conversion to AD in aMCI patients.

The study was supported by RSF grant 24-15-00220

**Disclosure of Interest:**

None Declared

